# The Structure of the Guanidine-II Riboswitch

**DOI:** 10.1016/j.chembiol.2017.05.014

**Published:** 2017-06-22

**Authors:** Lin Huang, Jia Wang, David M.J. Lilley

**Affiliations:** 1 Cancer Research UK Nucleic Acid Structure Research Group, MSI/WTB Complex, The University of Dundee, Dow Street, Dundee DD1 5EH, UK

**Keywords:** gene regulation, RNA structure, X-ray crystallography, riboregulation

## Abstract

The guanidine-II (mini-*ykkC*) riboswitch is the smallest of the guanidine-responsive riboswitches, comprising two stem loops of similar sequence. We have solved high-resolution crystal structures of both stem loops for the riboswitch from *Gloeobacter violaceus*. The stem loops have a strong propensity to dimerize by intimate loop-loop interaction. The dimerization creates specific binding pockets for two guanidine molecules, explaining their cooperative binding. Within the binding pockets the ligands are hydrogen bonded to a guanine at O6 and N7, and to successive backbone phosphates. Additionally they are each stacked upon a guanine nucleobase. One side of the pocket has an opening to the solvent, slightly lowering the specificity of ligand binding, and structures with bound methylguanidine, aminoguanidine, and agmatine show how this is possible.

## Highlights

• Guanidine-II riboswitch stem loops dimerize in the presence of the ligand
• Loop-loop interaction generates specific binding sites for two guanidine molecules
• Guanidine is hydrogen bonded to a guanine nucleobase, and to the phosphate backbone
• Some modified guanidine compounds also bind to the pocket, which is open on one side

## Graphical Abstract

**Figure:**
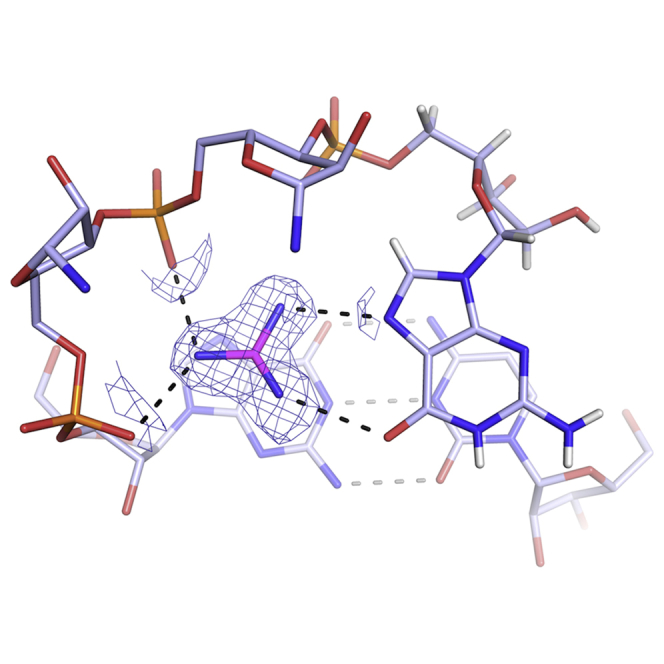

## Teaser

Huang et al. use structural biology to show that the stem loops of the guanidine-II dimerize by loop-loop interaction. Guanidine is hydrogen bonded to the Hoogsteen edge of a guanine that forms a base pair with a cytosine from the second stem loop. Guanidine stabilizes the structure to activate the gene.

## Introduction

Riboswitches are regulatory elements in mRNA that bind specific ligands, usually leading to the stabilization of a conformation that results in a changed level of gene expression (Roth and Breaker, 2009, Serganov and Nudler, 2013). The ligand will be metabolically related to the substrate of the enzyme or transporter that is the product of the gene in question, and expression is controlled at the level of transcription, translation, or mRNA stability. Riboswitches are widespread in bacteria, and many classes have now been identified, responding to a variety of metabolites including coenzymes, amino acids, purines, and even single ions. While the ligands for many riboswitches were straightforwardly identified, some have taken a considerable period to assign, requiring a rethinking of some aspects of cellular metabolism in the process.

One such is the bacterial *ykkC* element. This was identified as a candidate riboswitch in 2004 (Barrick et al., 2004), but only recently was the ligand identified as guanidine (Nelson et al., 2017). This required a re-evaluation of the role of this toxic compound in the cell (Breaker et al., 2017), and in some cases a correction of the annotation of some enzymes whose genes might be under the control of the putative riboswitch. The Breaker laboratory (Nelson et al., 2017) has shown that these riboswitches bind guanidine, leading to the upregulated expression of a series of genes whose products either chemically convert guanidine into other compounds or pump it from the cell. Due to its high p*K*_a_, guanidine will be protonated at physiological pH, thus existing as the positively charged guanidinium cation. For simplicity, herein we nevertheless in general refer to this as guanidine except where its charge is relevant.

Three classes of *ykkC* elements have been identified, now called the guanidine-I (Nelson et al., 2017), -II (Sherlock et al., 2017), and -III (Sherlock and Breaker, 2017) riboswitches. The first of these was shown to bind a molecule of guanidine with micromolar affinity, resulting in a change of conformation that sequestered part of the RNA required to form a transcription terminator stem loop, thus resulting in increased levels of transcription (Nelson et al., 2017). Two crystal structures have shown how the riboswitch is folded and the manner of the guanidine binding (Battaglia et al., 2017, Reiss et al., 2017). The guanidinium cation has D_3h_ symmetry and a positive charge. It is enclosed in a box of guanine nucleotides, such that all six protons make specific hydrogen bonds, and it is stacked onto the nucleobase of one guanine making a cation-π interaction. This pocket is highly selective for guanidine, excluding all similar compounds including urea.

**Figure 1:**
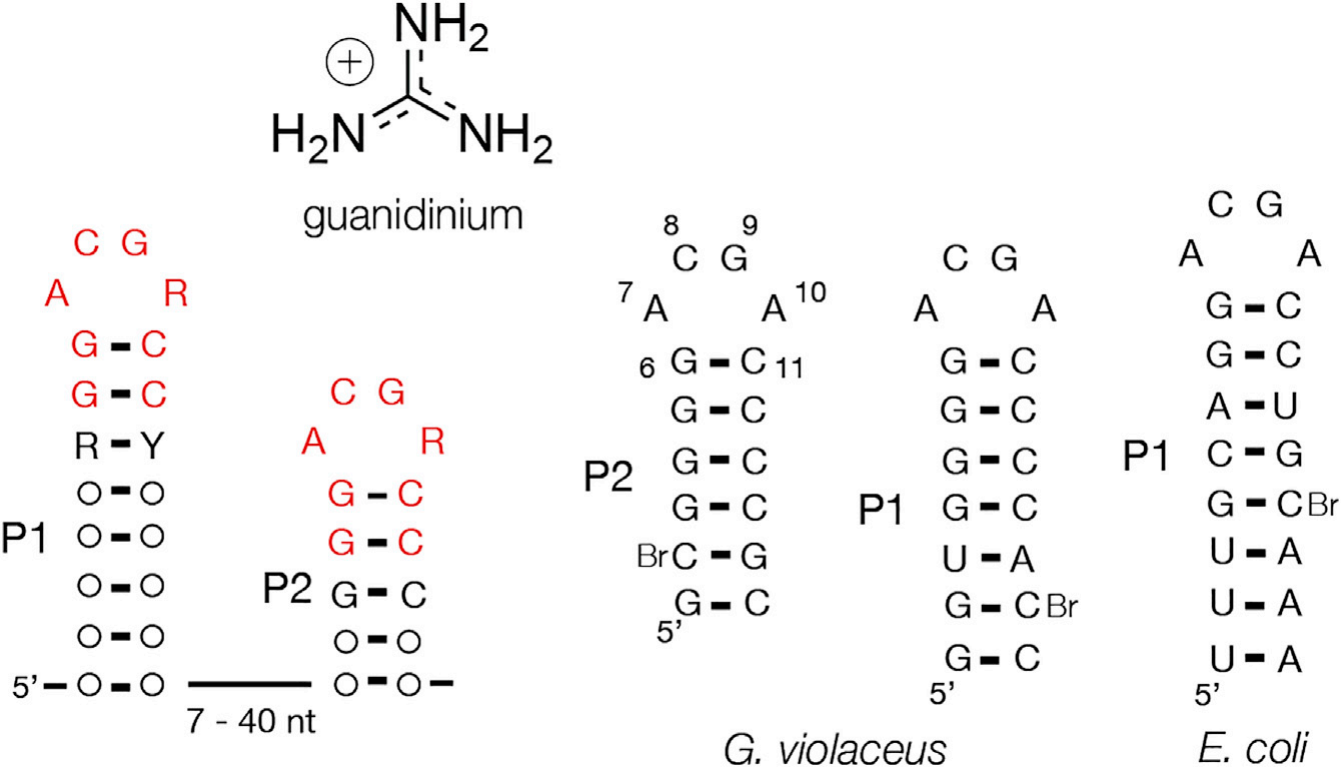
Sequences of the Guanidine-II Riboswitch Left: the consensus sequence of the riboswitch, with conserved sequences highlighted in red. R = A or G; Y = C or U. The ribozyme consists of two stem loops P1 and P2 of closely similar sequence, connected by a spacer sequence of variable length, although the great majority are ≥13 nt. The sequences of the P2 and P1 stem loops from the *G. violaceus* and the P1 stem loop of *E. coli* guanidine-II riboswitch are shown (right). Bromocytosine was included at the positions indicated (Br); this was used to phase the diffraction by its anomalous scatter. The numbering of the nucleotide positions of the P2 stem loop studied in this work is indicated. The structure of guanidine is drawn at the top, shown as it exists at neutral pH in its protonated form as the guanidinium cation.

Of the three types of guanidine riboswitch, the smallest (the guanidine-II riboswitch) comprises two stem loops connected by a short linking segment of polynucleotide (Sherlock et al., 2017) (Figure 1). The riboswitch was originally called the mini-*ykkC* motif, but we use the term guanidine-II riboswitch herein. Approximately 800 examples of this motif are known. These are located close to the ribosome binding sites of genes involved in guanidine metabolism, either efflux pumps (slightly more than half the examples) or modifying enzymes, e.g., carboxylases. Thus like the class I riboswitches they should act as ON switches (most probably by controlling translation of the mRNA) that lead to upregulation of genes involved in guanidine detoxification.

The sequences of the stem loops are highly conserved, comprising a G + C-rich helix and an ACGR (R = A or G) tetraloop. Upon addition of guanidine the two loop regions become protected in in-line probing experiments, indicative of a change of structure resulting from ligand binding (Sherlock et al., 2017). Alteration of the sequence of either one loop led to reduced protection of both loops, consistent with an interaction between them. Fitting the protection data gave a measurement of the affinity for guanidine of ∼50 μM, similar to that of the class I guanidine riboswitch. Importantly, the fit required a Hill coefficient of 1.4, consistent with the cooperative binding of two guanidine molecules, one to each loop. Moreover, in in-line probing experiments, Sherlock et al. (2017) showed that a single stem loop exhibited guanidine-induced protection at higher RNA concentrations, indicating that they could form homodimers that bind ligand. Given the high conservation of sequence this is perhaps not surprising; for example, the two stem loops of the *Gloeobacter violaceus* guanidine-II riboswitch are identical in their tetraloops (ACGA) and the adjacent four base pairs.

Sherlock et al. (2017) found that some variants of guanidine such as methylguanidine and aminoguanidine bound to the guanidine-II riboswitch with affinities that were within a factor of four of guanidine itself. The small size of the guanidine-II riboswitch makes it likely that its manner of binding the guanidine ligand will differ in some respects from the class I riboswitch, which employs five guanine nucleotides to surround and interact with the ligand, and this could result in a slightly lowered selectivity of ligand binding for the smaller riboswitch.

We therefore set out to determine crystal structures of the conserved stem loops of the *G. violaceus* guanidine-II riboswitch, with and without ligand, to investigate the structure, dimerization, and the manner of ligand binding. We find that all these stem loops do indeed dimerize by a loop-loop interaction, which explains all the nucleotide conservation that is the key to the function of the riboswitch. Loop-loop interaction creates two pockets for the binding of guanidine molecules resulting in cooperative binding, so that dimerization is central to the function of this riboswitch. The specificity of ligand binding is slightly lower than that for the guanidine-I riboswitch (Battaglia et al., 2017, Reiss et al., 2017), with fewer hydrogen bonds to the guanidine and an open side that allows for small variations in the ligand structure.

## Results

### Design and Synthesis

In this study we have solved crystal structures of several stem loops derived from guanidine-II riboswitches (Figure 1 and Table S1). These are the P2 and P1 stem loops from *G. violaceus* with and without bound ligand. All comprise GC-rich helices with ACGA terminal loops. All the RNA species were made by chemical synthesis, incorporating bromocytosine at the penultimate base pair of the stem, the anomalous scatter from which was used to provide phases for the diffraction. Each structure was solved by single-wavelength anomalous diffraction (SAD), except for the structures with modified ligands bound that were solved by molecular replacement. The crystallographic statistics are presented in Table 1. As discussed in detail below, each RNA forms a dimer in the crystal, associated by an intimate association between the loops.

**Table 1:** Details of Data Collection and Refinement Statistics for the Crystallographic Data as Deposited with the PDB

Type	*E. coli* P1_8bp	*G. violaceus* P1_7bp	*G. violaceus* P1_7bp	*G. violaceus* P1_7bp	*G. violaceus* P1_7bp	*G. violaceus* P1_7bp	*G. violaceus* P2_6bp
Ligands	guanidine	ammonium	guanidine	methylguanidine	aminoguanidine	agmatine	guanidine
PDB ID	5NDI	5NEO	5NOM	5NEP	5NEQ	5NEX	5NDH

**Data Collection**							

Space group	P 2_1_ 2_1_ 2_1_	H3_2_	H3_2_	H3_2_	H3_2_	H3_2_	P 2_1_ 2_1_ 2_1_
Cell dimensions							
*a*, *b*, *c* (Å)	47.5, 48.2, 153.1	55.3, 55.3, 130.2	55.7, 55.7, 132.6	55.5, 55.5, 132.4	55.5, 55.5, 130.3	55.6, 55.6, 135.93	48.7, 57.8, 100.9
*α, β, γ* (°)	90, 90, 90	90, 90, 120	90, 90, 120	90, 90, 120	90, 90, 120	90, 90, 120	90, 90, 90
	SAD-Br	SAD-Br	MR	MR	MR	SAD-Br	SAD-Br
	peak	peak				peak	peak
Wavelength	0.9201	0.9201	0.9201	0.9201	0.9201	0.9179	0.9201
Resolution (Å)	38.27–2.57 (2.61–2.57)	26.92–1.69 (1.72–1.69)	44.19–1.93 (1.96–1.93)	27.75–1.60 (1.63–1.60)	38.66–1.69 (1.72–1.69)	45.42–1.72 (1.75–1.72)	50.17–1.81 (1.84–1.81)
*R*_merge_	0.082 (1.459)	0.066 (1.814)	0.084 (1.751)	0.062 (3.10)	0.072 (2.55)	0.129 (3.91)	0.059 (1.523)
*I*/σ*I*	10.7 (1.1)	17.1 (1.6)	16.4 (1.3)	17.4 (1.2)	13.7 (1.3)	10.7 (1.4)	16.2 (1.1)
CC_1/2_	1.00 (0.50)	0.95 (0.95)	1.00 (0.56)	1.00 (0.92)	1.00 (0.87)	1.00 (0.75)	1.00 (0.55)
Completeness (%)	100 (99.3)	99.9 (99.8)	99.3 (100)	100 (99.1)	99.8 (99.5)	100 (99.8)	100 (98.6)
Redundancy	5.4 (5.3)	19 (18.5)	9.4 (9.6)	18.8 (19.1)	14.1 (13.4)	18.5 (17.8)	6.3 (6.4)

**Refinement**							

Resolution (Å)	38.27–2.57 (2.66–2.57)	26.92–1.69 (1.75–1.69)	44.19–1.93 (2.00–1.93)	27.26–1.60 (1.66–1.60)	27.73–1.69 (1.75–1.69)	45.42–1.72 (1.78–1.72)	50.17–1.81 (1.88–1.81)
No. of reflections	11,428 (1,089)	8,835 (860)	6,369 (617)	10,472 (976)	8,783 (820)	8,886 (872)	26,472 (2,543)
*R*_work_/*R*_free_	0.196/0.243	0.223/0.237	0.224/0.246	0.214/0.221	0.218/0.257	0.208/0.235	0.213/0.229
No. of atoms							
Macromolecules	1,616	364	364	364	364	364	1,288
Ligands	105	30	33	46	44	48	119
*B* factors							
Macromolecules	69.97	45.89	43.42	41.61	42.13	39.32	41.34
Ligands	68.04	49.74	55.98	50.89	72.01	47.77	74.88
Solvent	63.50	48.46	46.57	48.76	49.68	52.28	48.67
RMSDs							
Bond lengths (Å)	0.006	0.003	0.018	0.046	0.010	0.007	0.005
Bond angles (°)	1.05	0.68	1.98	1.33	1.60	1.25	0.94

Br, bromocytosine; MR, molecular replacement; SAD, single-wavelength anomalous diffraction.

Values in parentheses represent the highest-resolution shell.

### Basic Structure of the Guanidine-II Riboswitch P2 Stem Loop Bound to Guanidine

**Figure 2:**
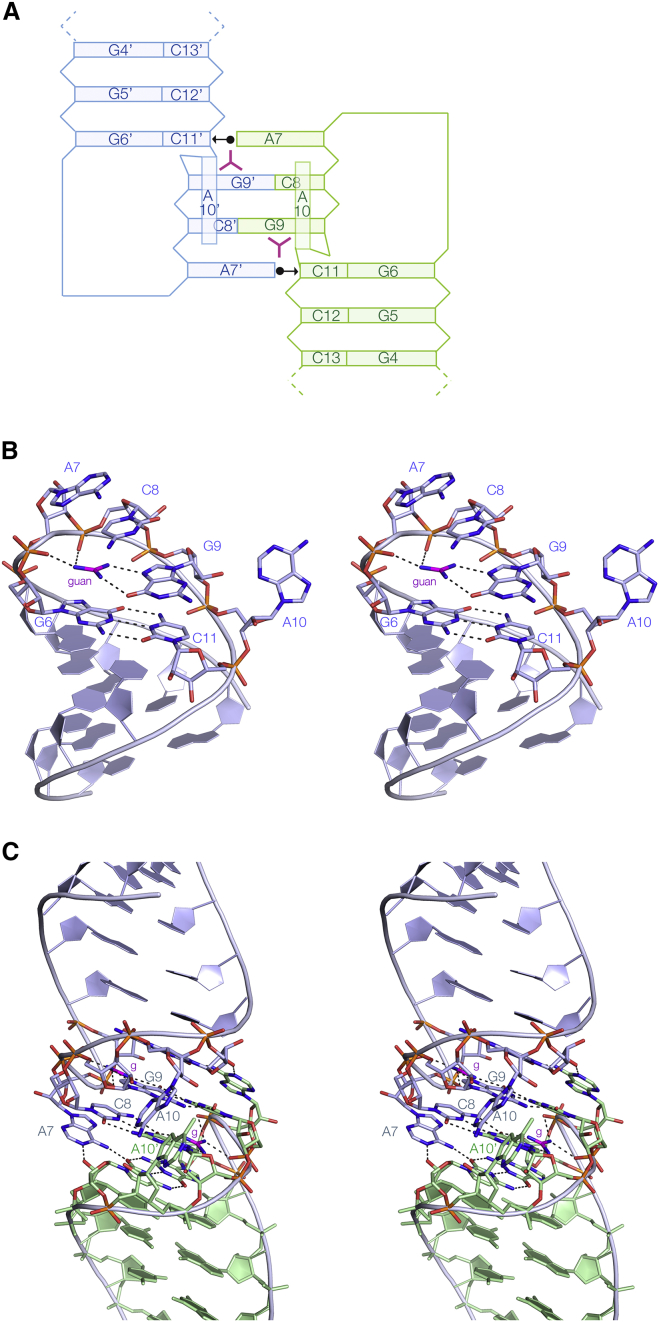
The Structure of the *G. violaceus* Guanidine-II Riboswitch P2 Stem Loop and Its Dimerization (A) Scheme illustrating the interactions between the two stem loops of the dimer. (B) A single P2 stem loop, with its dimeric partner removed for clarity. guan, guanidine (shown in magenta). (C) The dimeric complex of the P2 stem loop, with the two stem loops differentiated by color as blue and green. The nucleotides are numbered according to Figure 1, with those for the green stem loop distinguished by primes. g, guanidine (shown magenta). Broken lines depict hydrogen bonds throughout.

We have solved the structure of a 6-bp *G. violaceus* P2 stem loop bound to its guanidine ligand at 1.8-Å resolution (Figure 2; PDB: 5NDH). The crystals are orthorhombic (P2_1_2_1_2_1_), and two dimeric RNA complexes comprise the asymmetric unit (ASU). The structures of the crystallographically unrelated RNA molecules in the ASU superimpose with a root-mean-square deviation (RMSD) of 0.28 Å (in this and subsequent calculations we superimpose the loop and top two base pairs of the stem to calculate the all-atom RMSD). The duplex stems contain only C-G base pairs and are fully base paired (Figures 2B and S1). The helix is closed by the G6-C11 base pair, following which there is a very sharp turn in the trajectory of the backbone. The nucleobases of A7, C8, and G9 are stacked on each other, making interactions with nucleotides of the second loop in the dimer (see below). The nucleobase of A10 is extrahelical, after which C11 pairs with G6 to resume the stem.

### Dimerization of the Guanidine-II Riboswitch P2 Stem Loop

Each dimer within the ASU is created by a close association between the two ACGA terminal loops (Figures 2A and 2C), with almost coaxial alignment of the two stems. Breaker et al. (2017) had suggested that the two stem loops might interact by means of a “hand-to-wrist” architecture. The central C8 and G9 of each loop make complementary *cis* Watson-Crick base pairs, i.e., cross-strand C8-G9′ and G9-C8′ base pairs. A7 is coplanar with the closing G6′-C11′ base pair from the other stem loop, hydrogen bonding to the sugar edge of C11′, between A7N6-C11′O2 and A7N1-C11′O2′. At the other end of the loop A10 and A10′ nucleobases are extruded and inclined at 20° to the helical axis, and are mutually stacked with a spacing of 3.3 Å.

### The Manner of Guanidine Binding to the Guanidine-II Riboswitch P2 Stem Loop

**Figure 3:**
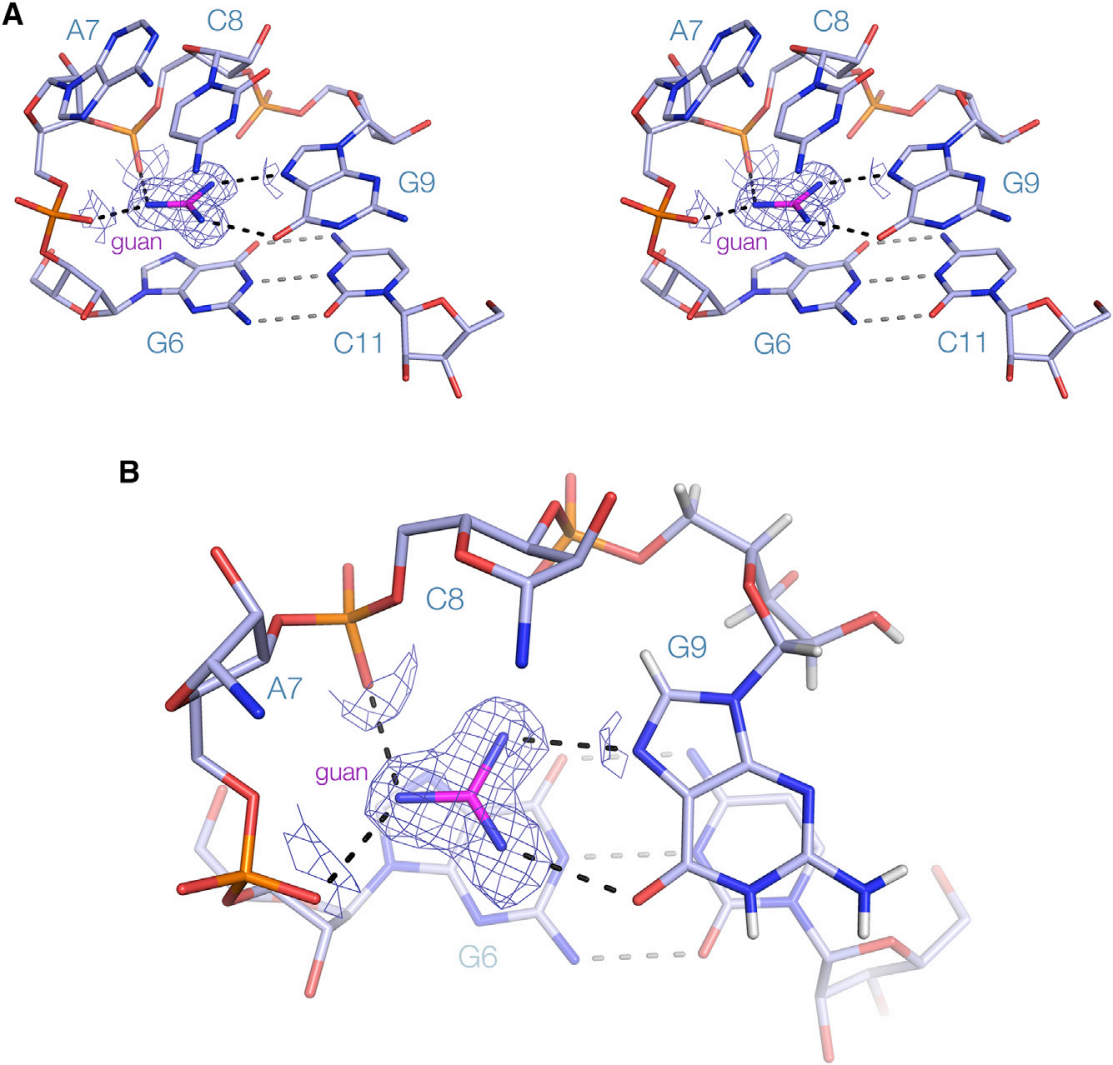
The Guanidine Binding Pocket of the *G. violaceus* Guanidine-II Riboswitch P2 Stem Loop, with the Second Stem Loop of the Dimer Removed for Clarity The unbiased electron density map (contoured at 1.2σ) is shown for the guanidine. (A) A parallel-eye stereoscopic view into the binding pocket. (B) A simplified view looking down onto the guanidine. Some nucleobases (A7 and C8) and backbone segments have been removed to give an unobscured view of the ligand binding.

The interacting loops of the dimer together create two binding sites for guanidine molecules (Figure 3). Each guanidine molecule binds coplanar with the G9-C8′ base pair, hydrogen bonded to G9 O6 and N7. The remaining amine of each guanidine is hydrogen bonded to the *pro*S and *pro*R non-bridging oxygen atoms of phosphates of A7 and C8, respectively, at the very tight turn of the loop. The distance between one nitrogen and the *pro*R oxygen of G9 is a little longer than a normal hydrogen bond and the geometry suboptimal, so we do not include this as one of the key interactions, although if there is some flexibility in the pocket this might become significant. The guanidinium cation is stacked upon the nucleobase of G6, making a cation-π interaction (Gallivan and Dougherty, 1999, Wintjens et al., 2000). The space above the guanidine is closed off by the nucleobases of C8 and A7, and thus the ligand is enclosed in a box, except for a space between the backbones of both loops at their tight turns, on the major groove side of the cross-strand C-G base pairs (Figure S2). The two guanidine molecules are vertically spaced by one base pair along the axis of the dimer, and separated by 9 Å.

### Structure of the Guanidine-II Riboswitch P1 Stem Loop with Bound Guanidine Ligand

We have solved the structure of a 7-bp *G. violaceus* P1 stem loop bound to its guanidine ligand at 1.9-Å resolution (Figure S3; PDB: 5NOM). The sequence is identical to that of P2 for the central 10 nt. However, this structure was solved in a trigonal space group (H3_2_) in which the ASU contains a single RNA stem loop. Notwithstanding, the structure is closely similar to that obtained for the P2 loop, forming a dimer by the same loop-loop interactions. The dimer superimposes with that determined for P2 with an RMSD = 0.20 Å (Figure S4). Since the P1 and P2 structures were solved in very different spacegroups, we can be confident that this structure is unaffected by lattice contacts. The binding pocket and manner of the interaction with guanidine are essentially identical to that observed for the P2 stem loop above (Figures S5B and S5C).

In addition, we have solved the structure of a guanidine-II riboswitch P1 8-bp stem loop from *Escherichia coli* bound to guanidine. This crystallized in spacegroup P2_1_2_1_2_1_ with two RNA dimers in the ASU, and diffracted to the lower resolution of 2.6 Å (PDB: 5NDI). The structure is closely similar to that of the *G. violaceus* RNA, with an RMSD of 0.28 Å.

### Structure of the Guanidine-II Riboswitch P1 Stem Loop with Alternative Bound Ligands

**Figure 4:**
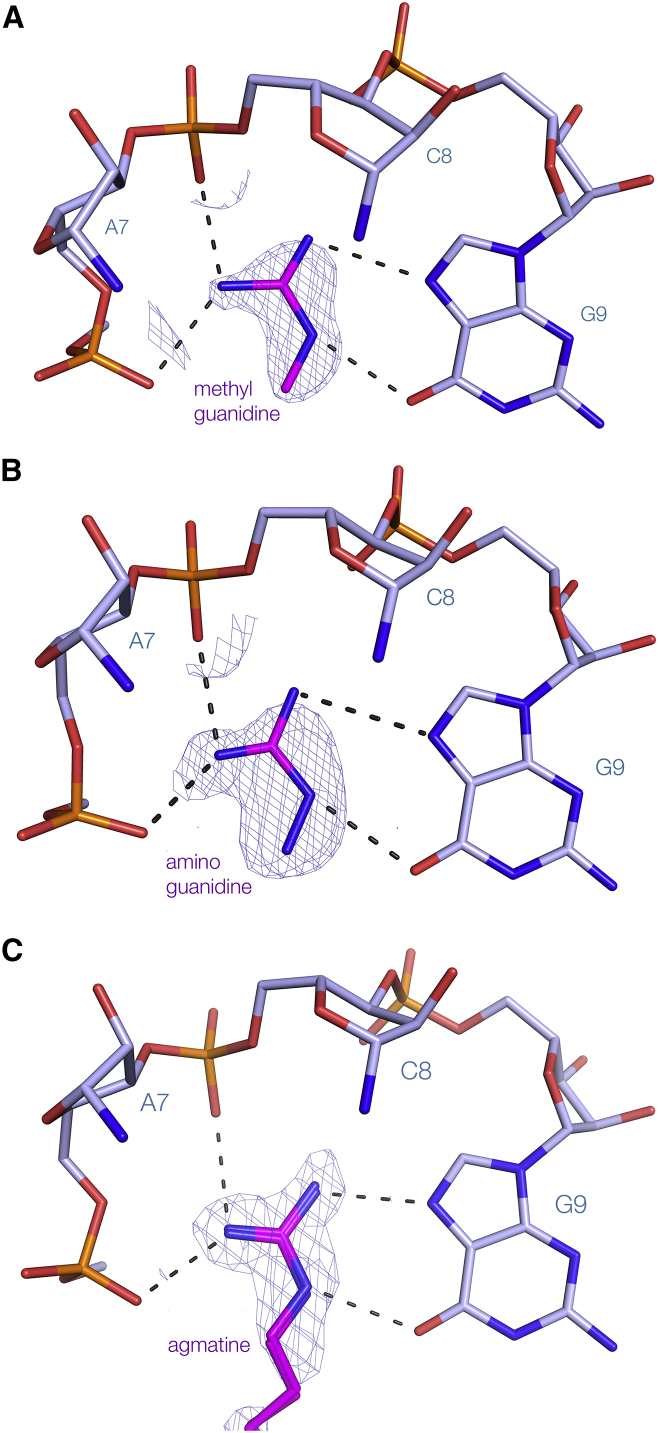
The Binding of Modified Analogs of Guanidine Bound to the *G. violaceus* Guanidine-II Riboswitch P1 Stem Loop (A) Methylguanidine, (B) aminoguanidine, and (C) agmatine. The composite omit map (contoured at 1.2σ) is shown for the methyl- and aminoguanidine analogs, and the unbiased electron density map for agmatine. Agmatine has been modeled in two conformations with equal occupancy. A more complete image of the binding pocket is shown in Figure S5D. In these images the nucleobases of A7 and C8 have been removed for clarity.

On the basis of in-line probing analysis, Breaker and colleagues (Sherlock et al., 2017) concluded that a number of small analogs of guanidine bound to the type II riboswitch, including methylguanidine and aminoguanidine. We therefore soaked crystals of ligand-free *G. violaceus* P1 stem loop with modified guanidine analogs (Table S1 and Figure S5A). Crystals of each were obtained in the same H3_2_ spacegroup, that diffracted to 1.7 Å or higher resolution, and the structures were solved by molecular replacement. In both cases the RNA structure was essentially identical to that with guanidine bound (RMSD = 0.13 and 0.10 Å for the methylguanidine-bound [PDB: 5NEP] and aminoguanidine-bound [PDB: 5NEQ] structures, respectively). Both ligands bind coplanar with G9, with adjacent nitrogen atoms hydrogen bonded to O6 and N7 (Figures 4A and 4B). The additional methyl and amino groups are attached to the nitrogen that is bound to G9 O6, directed to the open region between the two RNA backbones. We also obtained a structure of the P1 stem loop bound to agmatine (PDB: 5NEX), which has a butylamine side chain attached to one of the nitrogen atoms (Figures 4C and S5D). While the side chain has relatively weak electron density indicative of mobility, the guanidine moiety is observed to be bound in the same manner as the other ligands.

### Structure of the Guanidine-II Riboswitch P1 Stem Loop in the Absence of Guanidine Ligand

**Figure 5:**
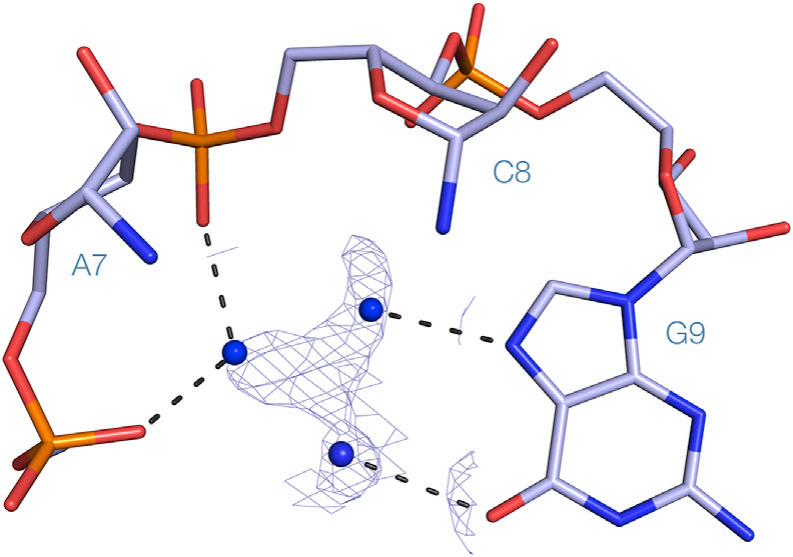
The Guanidine Binding Pocket *G. violaceus* Guanidine-II Riboswitch P1 Stem Loop in the Absence of Ligand The unbiased electron density map (contoured at 1.2σ) is shown in the pocket. This has been modeled as three atoms that are most probably ammonium ions. The nucleobases of A7 and C8 have been removed for clarity.

We have also solved the structure of the *G. violaceus* P1 stem loop bound in the absence of guanidine ligand at 1.69-Å resolution, in the same H3_2_ spacegroup. The RNA structure is essentially identical to that of the P1 stem loop with bound guanidine, with an RMSD of 0.32 Å (Figure S6; PDB: 5NEO). Interestingly, electron density corresponding to solvent is observed at the positions normally occupied by the amine groups of the guanidine (Figure 5). As these crystals formed only in the presence of a high concentration of (NH_4_)_2_SO_4_, these peaks most probably correspond to ammonium ions that make interactions with the RNA similar to those in the guanidine amino groups.

## Discussion

**Figure 6:**
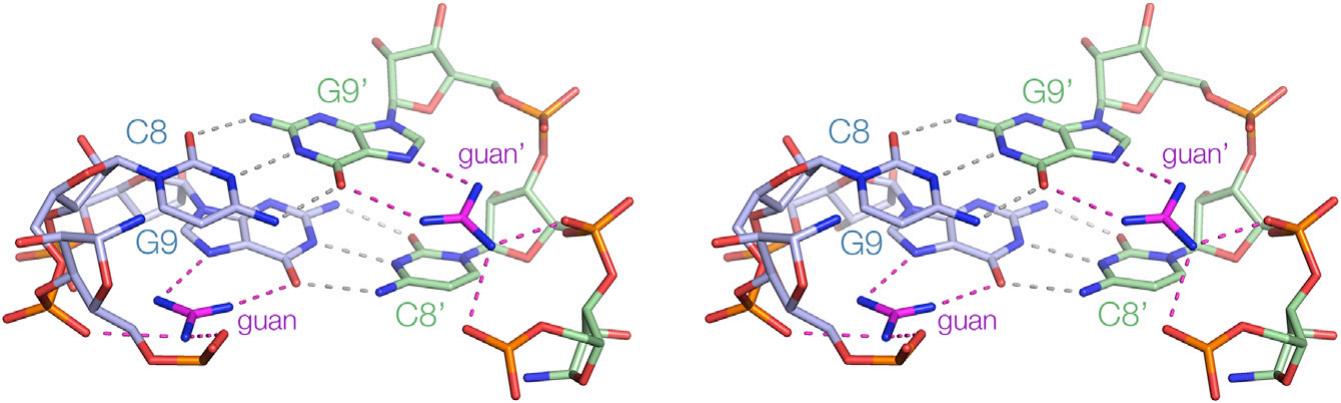
The Binding of Two Guanidine Molecules into the Pockets Created by Loop-Loop Interaction A parallel-eye stereoscopic view of the major groove side of the cross-strand C8-G9′ base pairs for the *G. violaceus* guanidine-II riboswitch P2 stem loops (differentiated by color as blue and green). Hydrogen bonds to the guanidine are highlighted in magenta. This view clearly shows that the two binding sites are shaped by the cross-strand base pairing between the two loops of the dimer.

Dimerization between the stem loops of the guanidine-II riboswitch is a critical aspect of their function. The loop-loop interaction creates the two specific ligand binding sites to which two guanidine molecules bind cooperatively (Figure 6). This results in guanidine binding with micromolar affinity, which is likely to be important for two reasons. First, any higher concentration is probably toxic to the cell, and so the genes required for detoxification must be turned on. Second, it should be higher than non-specific interaction of the cationic compound with RNA generally.

We have observed homodimerization of each guanidine-II riboswitch stem loop in significantly different space groups. Dimerization is clearly intrinsic to the nature of these structures, and is unlikely to differ significantly from heterodimerization between P1 and P2 stem loops in a complete riboswitch. The sequences of the loops and the helical stem adjacent to the loops are strongly conserved; in the case of the *G. violaceus* stem loops the sequence is identical for the loops (ACGA) and the adjacent four base pairs. Moreover, using in-line probing, Sherlock et al. (2017) showed that the pattern of reactivity for the *G. violaceus* P1 as a single stem loop exhibited exactly the same change on addition of guanidine as it did as part of the complete riboswitch with both stem loops. Since the loop-loop interaction results in collinear alignment of helical stems, the connecting segment must cover the full length of the two stems. For example, in the *G. violaceus* riboswitch a 14-nt linker must cover a 5′P to 3′P distance of 49 Å, and so will be well extended. Sherlock et al. (2017) have observed that on guanidine-induced folding of the riboswitch the linker region exhibits enhanced backbone scission by in-line probing.

Loop-loop interaction creates the specific guanidine binding pockets. Although all the direct contacts to a given guanidine are restricted to a single RNA loop, it is the central cross-strand base pairs between C8 and G9′ that direct the guanine nucleobases so that their Hoogsteen edges can interact with guanidine amine protons donating hydrogen bonds to O6 and N7 (Figure 6). This holds each guanidine so that it is juxtaposed with the backbone and can hydrogen bond to non-bridging oxygen atoms of successive phosphate groups. In addition, each guanidine is stacked upon the nucleobase of G6 in a cation-π interaction (Gallivan and Dougherty, 1999, Wintjens et al., 2000). In the absence of the loop-loop interaction it is likely that the individual loops are more flexible, giving an entropic penalty for binding the guanidine ligand. From the opposite perspective, binding guanidine will lower the free energy of the loop-loop interaction, thus stabilizing the dimeric conformation, which is the key to the riboswitch function.

The structures we present explain the strong conservation of the nucleotides in and around the loops of the guanidine-II riboswitches. The central C8pG9 forms the two cross-strand base pairs, which create the primary guanidine binding site at G9. A7 interacts with the sugar edge of C11′ (AN6 hydrogen bonded to C11′O2) and stacks on top of C8. This creates the tight turn of the backbone of the loop, presenting the phosphates to act as ligands for the guanidine. G6 (base paired with C11) provides the floor of the binding pocket on which the guanidine is stacked in the cation-π interaction (Gallivan and Dougherty, 1999, Wintjens et al., 2000). Lastly, the nucleotide at position 10 is conserved as either A or G. These are extrahelical and mutually stacked together; this interaction should be more stable for purine nucleotides.

The binding pocket has features in common with that of the guanidine-I riboswitch (Battaglia et al., 2017, Reiss et al., 2017), which also uses the Hoogsteen edge of a guanine nucleobase and the opposing backbone and stacks the guanidine on a guanine nucleobase. Binding of guanidino protons to O6 and N7 of a guanine nucleobase is also a recurring theme in RNA-protein interactions, for example in the use of arginine side chains by zinc-finger proteins (Pavletich and Pabo, 1991).

However, the binding pocket is not identical to that of the guanidine-I riboswitch. The latter is more enclosed, and all six protons of the guanidinium cation are potentially involved in hydrogen bonding. By contrast, the guanidine-II riboswitch has fewer direct interactions and a narrow opening on the major groove side of the cross-strand C-G pairs, and consequently the ligand selectivity is slightly lowered. We see that methylguanidine, aminoguanidine, and agmatine can be bound into the pocket, consistent with the biochemical experiments of Sherlock et al. (2017). Although the butylamine side chain of the latter has rather weak electron density, the guanidino group is well defined and the side chain emerges flexibly from the side opening. The binding site is evidently well disposed to binding compounds related to guanidine and closely related species, even organizing probable ammonium ions from the solvent within the pocket. However, the side opening is too narrow to accommodate anything more bulky than a methylene chain, consistent with the range of compounds found to fold the RNA biochemically (Sherlock et al., 2017).

In summary, we see how the dimerization of the guanidine-II riboswitch stem loops creates binding pockets for two guanidinium cations that stabilize the folded conformation of the RNA. In the *G. violaceus* riboswitch the P2 stem loop is 6 bp from the ribosome binding site (although the distance is variable in these riboswitches as a group), and a potential stem loop that overlaps both could form. The folded riboswitch and this putative stem loop would be mutually exclusive, so that stabilization of the riboswitch structure could expose the ribosome binding site. This would allow translation of the gene to proceed, i.e., upregulating the genes required to reverse guanidine toxicity in the cell.

## Significance

**Riboswitches are widespread regulatory elements in mRNA in bacteria that bind small-molecule ligands to affect local RNA conformation, leading to an altered level of gene expression. The guanidine riboswitches bind the guanidinium cation such that the level of expression of genes that deal with guanidine toxicity is upregulated. Three classes of guanidine riboswitches have been identified. The smallest of these (the guanidine-II riboswitch) comprises two connected stem loops, to which guanidine binds cooperatively. Crystal structures of these stem loops show that they are intrinsically disposed to dimerization by loop-loop interaction. Dimerization is key to the function of the riboswitch. The loop-loop interaction creates binding sites for two guanidine molecules, and thus the folded conformation of the RNA is stabilized by guanidine binding, which leads to the increased level of gene expression. Within the binding pockets the guanidinium cation is hydrogen bonded to the Hoogsteen edge of a guanine nucleobase that is base paired with the other RNA in the dimer. It is also hydrogen bonded with the backbone of the loop, and is stacked onto another guanine nucleobase in a cation-π interaction. However, one side of the binding pocket has a narrow opening that can accommodate a small side chain, and structures of the riboswitch reveal how compounds such as methylguanidine and aminoguanidine can be accommodated.**

## STAR★Methods

### Key Resources Table

**Table:** 

REAGENT or RESOURCE	SOURCE	IDENTIFIER
**Chemicals, Peptides, and Recombinant Proteins**		

Guanidine hydrochloride	Sigma-Aldrich	50993
Aminoguanidine hydrochloride	Sigma-Aldrich	396494
Methylguanidine hydrochloride	Sigma-Aldrich	222402
Agmatine sulfate	Sigma-Aldrich	A7127
5-bromocytidine	ChemGenes	ANP-5648
Triethylamine trihydrofluoride	Sigma-Aldrich	344648

**Deposited Data**		

Structure of *E. coli* P1_8bp with guanidine	This paper	5NDI
Structure of *G. violaceus* P1_7bp with ammonium	This paper	5NEO
Structure of *G. violaceus* P1_7bp with guanidine	This paper	5NOM
Structure of *G. violaceus* P1_7bp with methylguanidine	This paper	5NEP
Structure of *G. violaceus* P1_7bp with aminoguanidine	This paper	5NEQ
Structure of *G. violaceus* P1_7bp with agmatine	This paper	5NEX
Structure of *G. violaceus* P2_6bp with guanidine	This paper	5NDH

**Oligonucleotides**		

*E. coli* P1_8bp RNA sequence: UUUGCAGGACGACCUG(BrC)AAA	This paper	N/A
*G. violaceus* P1_7bp: GGUGGGGACGACCCCA(BrC)C	This paper	N/A
*G. violaceus_*P2_6bp: G(BrC)GGGGACGACCCCGC	This paper	N/A

**Software and Algorithms**		

Phenix	(Adams et al., 2010)	http://phenix-online.org
COOT	(Emsley et al., 2010)	https://www2.mrc-lmb.cam.ac.uk/personal/pemsley/coot/
XIA2 version 0.4.0.0	(Winter, 2010)	https://xia2.github.io
PDB_REDO	(Joosten et al., 2014)	http://www.cmbi.ru.nl/pdb_redo/

### Contact for Reagent and Resouces Sharing

Further information and requests for resources and reagents should be directed to and will be fulfilled by the Lead Contact, Professor David M. J. Lilley FRS (d.m.j.lilley@dundee.ac.uk)

### Experimental Model and Subject Details

All RNA used in our crystallographic studies was made by chemical synthesis. No animals or cell lines have been used in this work.

### Method Details

#### RNA Synthesis

RNA oligonucleotides were synthesized using *t*-BDMS phosphoramidite chemistry (Beaucage and Caruthers, 1981) as described in Wilson et al. (Wilson et al., 2001), implemented on an Applied Biosystems 394DNA/RNA synthesizer. Oligoribonucleotides containing 5-bromocytidine (ChemGenes) were deprotected in a 25% ethanol/ammonia solution for 36 h at 20°C. All oligoribonucleotides were redissolved in 115 μL of anhydrous DMSO, 60 μl triethylamine (Aldrich) and 75 μL triethylamine trihydrofluoride (Aldrich) to remove *t*-BDMS groups, and agitated at 65°C in the dark for 2.5 h. After cooling on ice for 10 min, 250 μL RNA quenching buffer (Glen Research) was added to stop the reaction and the oligonucleotides were desalted using NAP-10 columns (GE Healthcare).

RNA was further purified by gel electrophoresis in polyacrylamide under denaturing conditions in the presence of 7 M urea. The full-length RNA product was visualized by UV shadowing. The band was excised and electroeluted using an Elutrap Electroelution System (GE Healthcare) into 45 mM Tris-borate (pH 8.5), 5 mM EDTA buffer for 8 h. at 200 V at 4°C. The RNA was precipitated with ethanol, washed once with 70 % ethanol and dissolved in double-distilled water.

#### Chemicals and Reagents

Guanidine, methylguanidine and aminoguanidine were used as hydrochlorides, and agmatine as sulfate. All were purchased as the highest available grade from Sigma-Aldrich. Details are listed in the Key Resources Table.

#### Crystallization, Structure Determination, and Refinement

RNA sequences are shown in Figure S1 and Key Resources Table. A solution of 1 mM RNA in 5 mM HEPES (pH 7.6), 100 mM KCl was heated to 95 °C for 1 min. The solution was slowly cooled to 20°C and MgCl_2_ added to a final concentration of 2 mM. Guanidine was added to a final concentration of 10 mM. Other ligands were soaked into crystals of *G. violaceus* P1 ligand-free RNA using the conditions indicated in Table S1. All the crystals were cryoprotected using mother liquid with an additional 25-30% glycerol.

Diffraction data were collected on beamline I04 and I24 of Diamond Light Source (Harwell, UK) except for *G. violaceus* P1 with agmatine for which data were collected on beamline ID23-1 at the European Synchrotron Radiation Facility (Grenoble, France). Data were processed by XIA2 version 0.4.0.0 (Winter, 2010). The resolution cutoff for the data was determined by examining by CC1/2 and density map as described previously (Karplus and Diederichs, 2012). Initial phase information for structures 5NDI, 5NEO, 5NEX and 5NDH were acquired from the SAD data by locating the bromine atoms with Autosol in the PHENIX suite. Structures 5NOM, 5NEP and 5NEQ were determined by molecular replacement use the 5NEO as the initial model. Models were adjusted manually using Coot (Emsley et al., 2010) and subjected to several rounds of adjustment and optimization using Coot, phenix.refine and PDB_REDO (Joosten et al., 2014). Model geometry and the fit to the electron density maps were monitored with MOLPROBITY (Chen et al., 2010) and the validation tools in Coot. The unbiased electron density maps were generated through Br-SAD phasing and density modification by Phenix AutoSol. The composite omit map also generated by Phenix.

### Data and Software Avaliability

All software were reported in Method Details and indicated in the Key Resources Table.

The accession numbers for the coordinates and structure factors of all structures in this paper have been deposited in the PDB indicated in the Key Resources Table. They are as follows: 5NDI for structure of *E. coli* P1_8bp with guanidine; 5NEO for structure of *G. violaceus* P1_7bp with ammonium; 5NOM for structure of *G. violaceus* P1_7bp with guanidine; 5NEP for structure of *G. violaceus* P1_7bp with methylguanidine; 5NEQ for structure of *G. violaceus* P1_7bp with aminoguanidine; 5NEX for structure of *G. violaceus* P1_7bp with agmatine; and 5NDH for structure of *G. violaceus* P2_6bp with guanidine.

## Author Contributions

L.H., J.W., and D.M.J.L. planned experiments; L.H. and J.W. performed crystallography; L.H. and D.M.J.L. analyzed data and wrote the paper.

## Supplemental Information

Document S1. Figures S1–S6 and Table S1

Document S2. Article plus Supplemental Information
