# Supplementary material for: The Structure of the Guanidine-II Riboswitch
Source: Cell Chem Biol. 2017 Jun 22;24(6):695–702.e2. doi: 10.1016/j.chembiol.2017.05.014 (PMC5486947; doi:10.1016/j.chembiol.2017.05.014)
Supplement: Document S1. Figures S1–S6 and Table S1 [file mmc1.pdf]

Cell Chemical Biology, Volume *24*

Supplemental Information

The Structure of the Guanidine-II Riboswitch

Lin Huang, Jia Wang, and David M.J. Lilley

The structure of the guanidine-II riboswitch

L. Huang, J. Wang and D. M. J. Lilley

Supplementary Information

Supplementary Figures

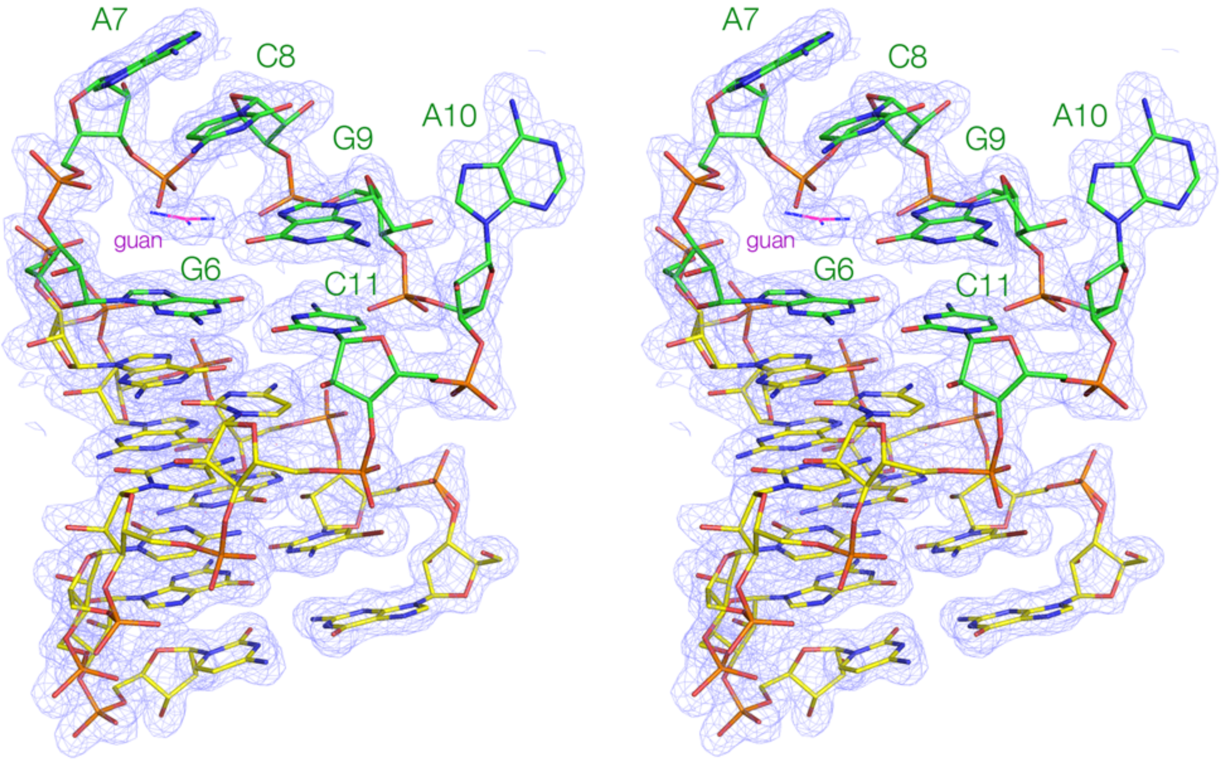

Figure S1. A parallel-eye stereoscopic view of the unbiased electron density map of single *G. violaceus* guanidine-II riboswitch P2 stem-loop. Linked to Figure 2.

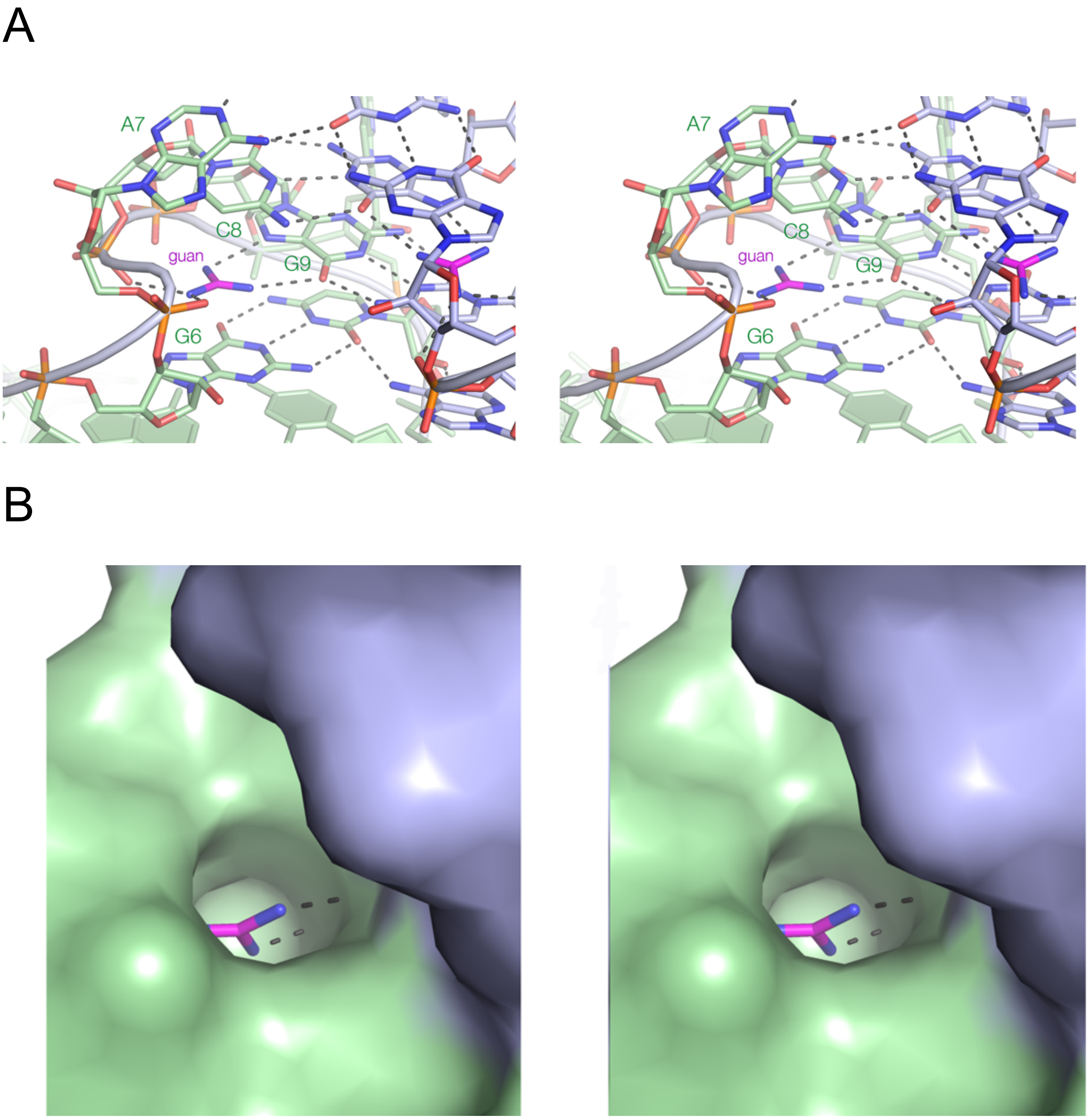

Figure S2. A parallel-eye stereoscopic view of the *G. violaceus* guanidine-II riboswitch P2 ligand binding pocket viewed from the open side. **A**. stick representation. **B**. A surface has been calculated for the RNA. The guanidine ligand (drawn in stick form, magenta) is visible through an open pore on this face. Note that these views of the RNA show the opposite face to that presented in Figure 2C. Linked to Figures 2 and 4.

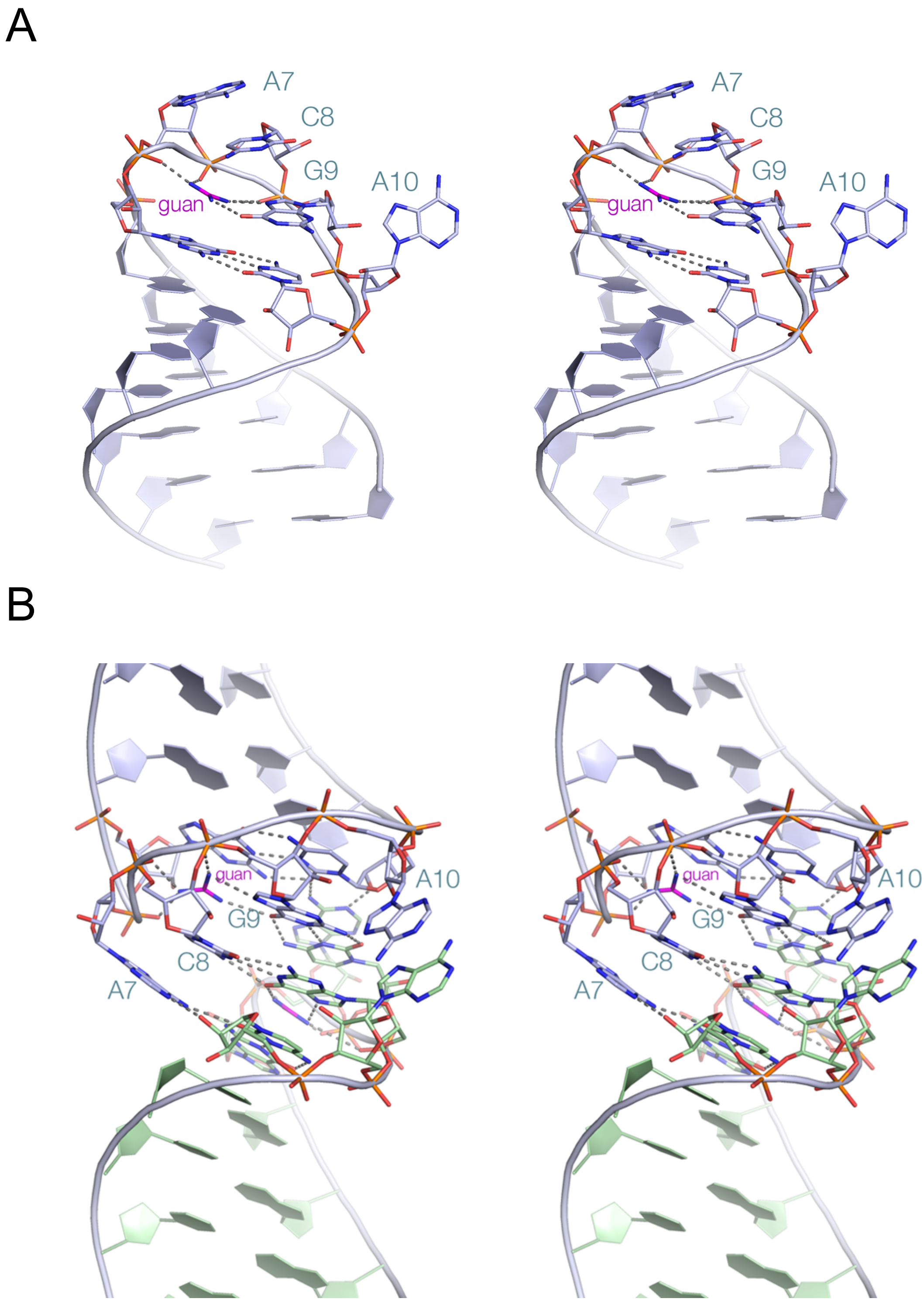

Figure S3. A parallel-eye stereoscopic views of the structure of *G. violaceus* guanidine-II riboswitch P1 stem-loop with bound guanidine. The numbering system for the nucleotides has been chosen to be directly comparable with that for the P2 stem-loop.

A. One stem-loop, with its dimeric partner removed for clarity

B. The dimer of stem-loop.

Linked to Figure 2.

A

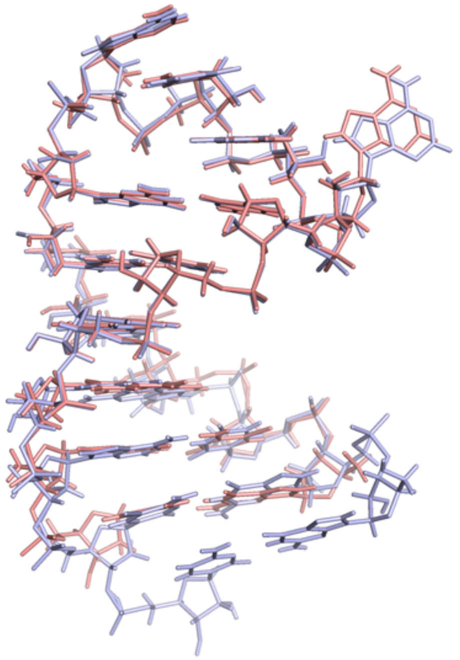

B

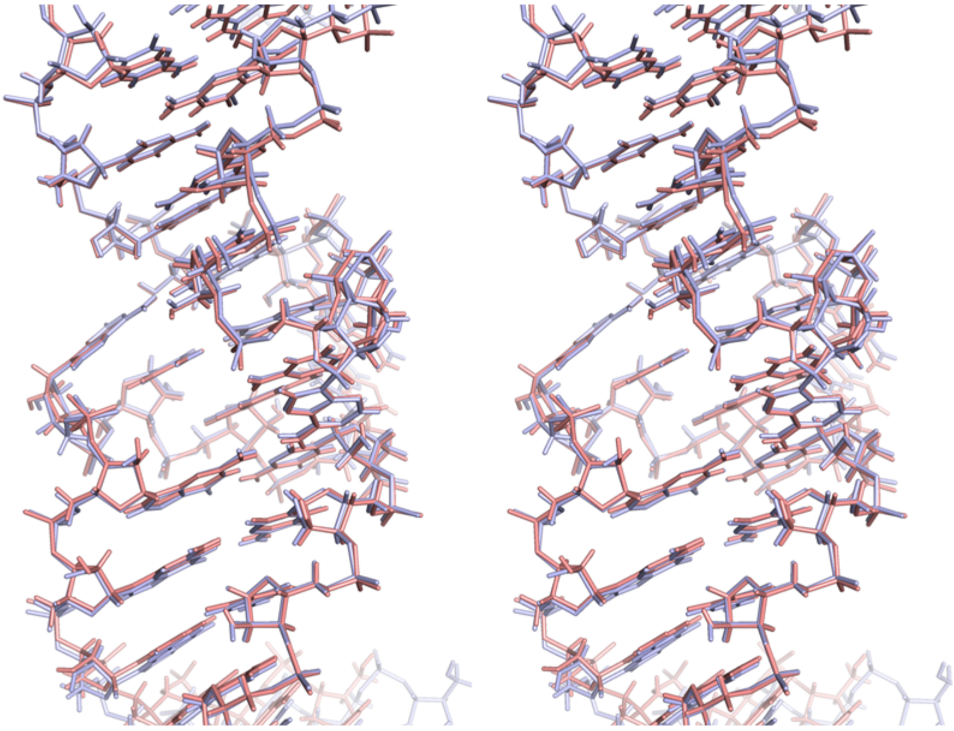

Figure S4. Parallel-eye stereoscopic views of superposed structures of the P1 and P2 stem-loops of *G. violaceus* guanidine-II riboswitch with bound guanidine.

A. As single stem-loops

B. As dimeric complexes. P1 is shown blue and P2 shown red.

Linked to Figure 2.

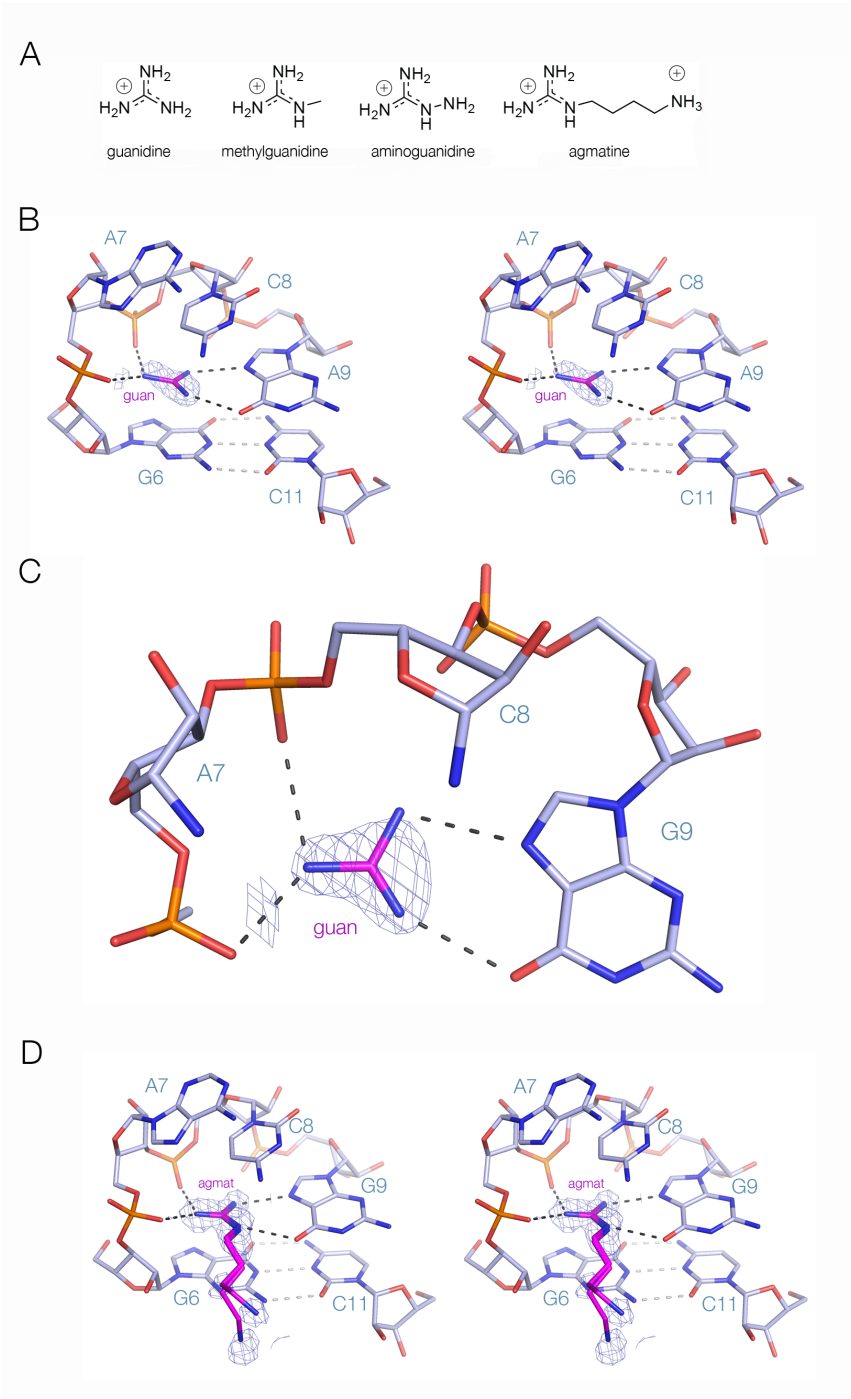

Figure S5. The guanidine binding pocket of the *G. violaceus* guanidine-II riboswitch P1 stem-loop.

A. The structure of the four ligands used in this study.

B. A parallel-eye stereoscopic view into the binding pocket with guanidine bound. The unbiased electron density map (contoured at 1.2 σ) is shown for the guanidine.

C. A simplified view looking down onto the guanidine. Some nucleobases (A7 and C8) and backbone segments have been removed for clarity.

D. A parallel-eye stereoscopic views of the structure of *G. violaceus* guanidine-II riboswitch P1 stem-loop with bound agmatine. The unbiased electron density map (contoured at 1.2 σ) is shown for the agmatine. The ligand is shown in two conformations with equal occupancy.

Linked to Figure 4.

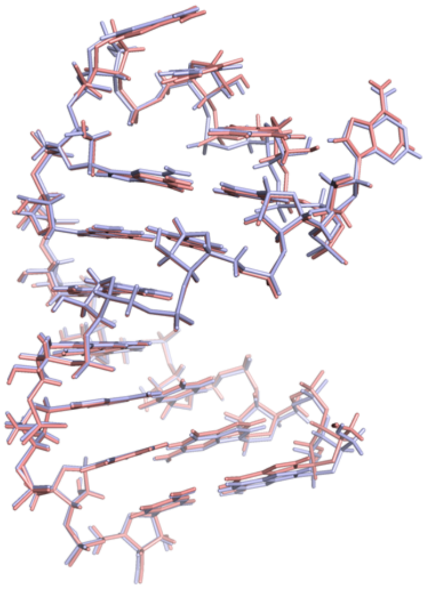

Figure S6. Parallel-eye stereoscopic views of superposed structures of the P1 and P2 stem-loops of *G. violaceus* guanidine-II riboswitch with (blue) and without (red) bound guanidine. Linked to Figures 2 and 4.

Supplementary Tables

PDB	RNA	ligands	mother liquor	r	Resol Å	space group	beamline
5NDI	*E. coli* P1_8bp	cocrystallized 10 mM guanidine	0.01 M magnesium chloride, 0.05 M MES pH 5.6, 2.0 M lithium sulfate monohydrate	20	2.57	$P2_12_12_1$	Diamond I04
5NEO	*G. violaceus* P1_7bp	cocrystallized 2.5 M ammonium	0.01 M magnesium acetate, 0.05 M MES pH 5.6, 2.5 M ammonium sulfate	20	1.69	$H3_2$	Diamond I04
5NEF	*G. violaceus*_ P1_7bp	cocrystallized 10 mM guanidine	0.15 M KCl 25% v/v Glycerol 0.05M Bis-Tris pH 7.0 1.6M ammonium sulfate	7	1.91	$H3_2$	Diamond I24
5NEP	*G. violaceus* P1_7bp	soaked 100 mM methylguanidine	0.01 M magnesium acetate, 0.05 M MES pH 5.6, 2.5 M ammonium sulfate	20	1.60	$H3_2$	Diamond I24
5NEQ	*G. violaceus* P1_7bp	soaked 10mM aminoguanidine	0.01 M magnesium acetate, 0.05 M MES pH 5.6, 2.5 M ammonium sulfate	20	1.69	$H3_2$	Diamond I24
5NEX	*G. violaceus* P1_7bp	soaked 500 mM agmatine	0.01 M magnesium acetate, 0.05 M MES pH 5.6, 2.5 M ammonium sulfate	20	1.72	$H3_2$	ESRF ID23-1
5NDH	*G. violaceus* P2_6bp	Cocrystallized 10 mM guanidine	0.01 M magnesium Sulfate, 0.05 M sodium cacodylate pH 6.0, 1.8 M lithium sulfate monohydrate	20	1.81	$P2_12_12_1$	Diamond I04

Table S1. Summary of the RNA species, ligands and crystallization conditions used in these experiments, and the crystals obtained. Linked to Table 1.
